# A comprehensive study on maxillofacial trauma conducted in Yamunanagar, India

**DOI:** 10.5249/jivr.v5i2.331

**Published:** 2013-06

**Authors:** Rishi Bali, Parveen Sharma, Amandeep Garg, Guneet Dhillon

**Affiliations:** ^*a*^Department of OMFS, D.A.V. Dental College and M.M. General Hospital, Yamunanagar – 135001,India.

**Keywords:** Maxillofacial trauma, Fractures, Clinical Study

## Abstract

**Background:**

The Department of Oral and Maxillofacial Surgery, D.A.V [C] Dental College and Hospital, Yamuna Nagar, Haryana, India conducted a study on patients with maxillofacial fractures in a time span of seven years (2003-2010). The purpose of this study was to evaluate their aetiology, incidence, patterns and different modalities employed for management.

**Methods:**

In this study, 740 patients with 1054 fractures were evaluated clinically and radiographically, based on which closed reduction and open reduction was undertaken. Review of patient records included: Age, sex, time, mechanism and etiology of injury, history of bleeding, unconsciousness and prior first aid, type of vehicle and use of preventive measures, type of fracture and treatment modalities.

**Results:**

Road traffic accidents accounted for highest number of fractures predominantly occurring in the age group of 21-30 years (38.3%)1,2. Males incurred more fractures with a male female ratio of 4.2: 1.Mandible was the most commonly fractured bone with parasymphysis being the commonest affected site.76.66% patients had associated head injury and 15.68 % had history of unconsciousness. Open reduction and internal fixation was the preferred modality for mandible whereas the mid face fractures were treated more often by closed methods.

**Conclusions:**

Injuries occurred more commonly in 20 – 40 age range with road traffic accident being the major etiological factor. Majority of the patients were driving two wheelers and most were under the effect of alcohol. Most of the injuries occurred during night and road traffic accidents (71.89%) were found to be the major etiological factor. Out of 532 road traffic accidents, 490 patients (66.2%) were on two wheelers, among whom 49(10%) were wearing helmet. In the mandible, fractures occurred most commonly in the parasymphyseal region (224, 30.2%), and out of the 314 fractures of the middle third showed, 155 (49.4%) ZMC. OPG was the most commonly advised X-ray. With regard to treatment modalities, 36.8% of all the mandibular fractures (740) were treated by closed reduction, 62.6% were treated using open reduction and 0.5% was under observation only.

## Introduction

Maxillofacial region (MFR) involves soft and hard tissues forming the face extending from frontal bone superiorly to the mandible inferiorly. The face being the most exposed part of the body is particularly prone to trauma. Trauma to the facial region causes injuries to skeletal components, dentition as well as soft tissues of the face. Injuries to the maxillofacial region are increasing in frequency and severity because of the heavy reliance on road transportation and the increasing socioeconomic activities of the population.^1-3^

Every 30 seconds someone dies on the world’s roads. Annually over 1 million people die and over 25 million are injured or permanently disabled from road traffic injuries.^4^ The primary cause of maxillofacial fractures throughout the world is road traffic accidents and assaults.^5^ Telfer MR et al (1991)^6^ conducted a study in United Kingdom, and reported that total number of patients with facial bone fractures had risen from 79 per annum in 1977 to 94 per annum in 1987, an increase of 20% which was highly significant, statistically. Number of patients injured in RTA had decreased by 34% while number of patients injured in assaults had increased by 47%. Other causes of maxillofacial fractures include falls, hit by animal, work related and sports related injuries.

In India inspite of the great impact of maxillofacial traumatic injuries on the patient’s quality of life, there is inadequate information about the epidemiological characteristic of this problem. In this backdrop, the present study has been undertaken to evaluate (i) the etiology of trauma and incidence of fractures according to the cause and establish relationship between the cause and fracture pattern. (ii) The increase in incidence of trauma due to alcohol intake. (iii) The incidence of fractures with respect to two wheelers and four wheelers.(iv) The pattern of maxillofacial injuries sustained by helmet and nonhelmeted motorcyclists in cases of two wheelers and in those wearing seat belts in case of four wheelers.(v) To identify anatomical site of fracture and associated injuries, and (vi) to evaluate different modalities of treatment rendered.

## Methods

The patients with maxillofacial fractures managed in the Department of Oral and Maxillofacial Surgery, D.A.V [C] Dental College and Hospital, Yamuna Nagar, Haryana, India in a time span of seven years (2003-2010) were selected for the study. All patients were treated irrespective of age, sex, caste, religion and socio-economic status. Patients were evaluated for any maxillofacial fracture by assessing clinically the displacement of fractured fragments, functional and cosmetic deficits, patient's age and patient's medical status. Exact determination of the site and pattern of bony injury was determined by correlating it radiographically using any of the following radiographs and CT scan as per indication The parameters on which patients were evaluated included.

1. Age of patient, 2. Gender distribution of patient, 3. Time of injury, 4. Etiology of fracture, 5. Mechanism of injury, 6. Type of vehicle, 7. Type of passenger (driver/pillion rider), 8. Use of helmet or seat belts, 9. Under the effect of alcohol or drugs, 10. History of bleeding, 11. History of unconsciousness, 12. Any prior first aid, 13. X-rays advised, 14. Site of fracture, 15. Associated injuries and 16. Treatment modalities.

The significances of the findings were evaluated using Pearson Chi-Square test . 

## Results

The 740 patients with 1054 maxillofacial fractures, managed in the Department of Oral and Maxillofacial Surgery from August 2003 to July 2010, were divided into 7 age groups. (Table 1,2,3)

**Table 1:** Group wise distribution of patients

Group-I	1 month-10 years	Group-V	41-50 years
Group-II	11-20 years	Group-VI	51-60 years
Group-III	21-30 years	Group-VII	61 years & above
Group-IV	31-40 years		

**Table 2:** Age wise distribution of patients

Age group (years)	Number of patients	Percentage
0-10	45	6%
11-20	145	19.7%
21-30	284	38.3%
31-40	150	20.2%
41-50	75	10%
51-60	30	4%
61 yrs and above	10	1.3%

**Table 3:** Gender wise distribution of patients

Gender	Number of patients	Percentage
Male	600	81.08%
Female	140	18.9%

Out of total 740 patients with maxillofacial fractures, 600 were males (81.08%) as against 140 females (18.9%), giving a male to female ratio of 4.2:1. (Table 4)

**Table 4:** Time of injury

Time	Number of patients	Percentage
Morning	186	25.13%
Mid day	198	26.75%
Midnight	356	48.1%

Most of the injuries occurred at night (48.1%). Injuries occurred at mid day and morning with almost equal frequency (26.75%) and (25.13%) respectively.(Table 5)

**Table 5:** Mechanism of injury

Mechanism of Injury	Number of patients	Percentage
Static Individual	69	9.3%
Moving Individual	369	49.9%
Combination	302	40.8%

Based on statistical analysis (Chi-Square Tests) it was concluded that majority of the injuries occurred during night (48.1%), and based on Lindahl’s classification of mechanism of injury,^7^ 9.3% patients were static individuals, 49.9% patients were moving and 40.8% patients were under combination group. (Table 6)

**Table 6:** Etiology of fractures

Etiology	Number of patients	Percentage
RTA	532	71.89%
• 2 wheeler	490	66.2%
• 4 wheeler	28	3.8%
• Pedestrian	14	1.9
Assault	42	5.6%
Fall	120	16.2%
Sports	21	2.8%
Miscellaneous	35	4.7%

Among the various etiological factors responsible for maxillofacial fractures, road traffic accidents (71.89%) were found to be the major etiological factor. (Table 7)

**Table 7:** Type of passengers

Vehicle (No.)	Injured patients (No.)	Percentage
2- wheeler (490)	Driver (374)	76.3%
	Pillion rider (116)	23.7%
4- wheeler (28)	Driver (15)	53.6%
	Front seater (4)	14.3%
	Rear seater (9)	32.1%

Out of 532 road traffic accidents, 490 patients (66.2%) were on two wheelers, 28 patients (3.8%) on four wheelers and 14 (1.9%) were pedestrians. Out of these 490 patients on two wheelers, 374 patients (76.3%) were drivers and 116 (23.7%) were pillion riders. Out of 28 patients on four wheelers, 15 patients (53.6%) were drivers, 4 (14.3%) were front seaters and 9 (32.1%) were rear seaters. Out of 490 patients driving two wheelers, 49(10%) were wearing helmet. (Table 8)

**Table 8:** Use of helmets/ seat belts

Vehicle (No.)	Used helmets/seat belt	Percentage
2- wheeler (490)	49	10%
4- wheeler (28)	9	32.14%

None of the pillion rider was wearing helmet. Out of 28 patients driving four wheelers, 9 (32.14%) were wearing seat belts. None of the front seater or rear seater passengers was wearing seat belts. (Table 9)

**Table 9:** Under the effect of alcohol

Effect of Alcohol	Number of patients	Percentage
Drunk	26	3.5%
Non drunk	714	96.48%

Among the 740 patients, 26 (3.5%) were under the effect of alcohol. (Table 10)

**Table 10:** History of bleed

History of bleed	Number	Percentage
Oral bleed	664	89.7%
Nasal bleed	59	7.9%
Ear bleed	14	1.9%
No bleed	3	0.4%

664 patients reported history of oral bleed (89.7%), 59 patients (7.9%) reported nasal bleed and 14 (1.9%) had ear bleed. 0.4% patients (3) had no history of oral, nasal or ear bleed. (Table 11)

**Table 11:** Neurological status

Neurological status	Number of patients	Percentage
Conscious	624	84.32%
H/o of Unconscious	116	15.68%

Out of total 740 patients, 116 patients (15.68%) reported history of unconsciousness.(Table 12)

**Table 12:** Prior First aid

Prior First aid	Number of patients	Percentage
Taken	589	79.5%
Not taken	151	20.4%

Among 740 patients of maxillofacial fractures, total 1054 fractures occurred including 117 dentoalveolar fractures (11.1%), average 1.4 fractures per patient. The site distribution of the fractures showed 740 fractures in the mandible (70.2%) including dentoalveolar fractures. Rest 314 (29.8%) fractures were distributed in rest of the maxillofacial skeleton. (Table 13)

**Table 13:** Sites of fracture

Facial bone	Number of fractures	Percentage
Mandible	706	66.9%
Middle third	223	21.2%
Dentoalveolar	117	11.1%

In the mandible, fractures occurred most commonly in the parasymphyseal region (224, 30.2%), followed by condylar region (213, 28.78%). Among the condylar fractures 6.2% (46), were bilateral whereas 16.4% (121) were unilateral condylar fractures. Third most common site for the fractures was angle (147, 19.9%), followed by body (72, 9.7%), dentoalveolar (34, 4.5%) and symphysis (12, 1.6%). Least common fractures reported were ramal fractures (6, 0.8%) and coronoid fractures (6, 0.8%). (Table 14)

**Table 14:** Fracture distribution in mandible

Fracture site	Number of fractures	Percentage
Condyle	213	28.78%
• Unilateral	121	16.4%
• Bilateral	46	6.2%
Coronoid	6	0.8%
Ramus	6	0.8%
Angle	147	19.9%
Body	72	9.7%
Parasymphysis	224	30.2%
Symphysis	12	1.6%
Dentoalveolar	34	4.5%

The site distribution of the 314 fractures of the middle third showed, 155 (49.4%) ZMC fractures, 80 (28.3%) maxillary dentoalveolar fractures, 38 (12.1%) Le Fort II, 19 (6.05%) Le Fort I, 11 (3.5%) Le Fort III, 3 (0.95%) Palatal split, 4 (1.3%) nasal and 1 (0.3%) NOE fractures. (Table 15,16)

**Table 15:** Fracture distribution in middle third

Fracture site	Number of fractures	Percentage
Le Fort I	19	6.05%
Le Fort II	38	12.1%
Le Fort III	11	3.5%
ZMC	155	49.4%
Dentoalveolar	83	28.3%
Palatal split	3	0.95%
NOE	1	0.3%
Nasal	4	1.3%

**Table 16:** Fracture site according to etiology

Etiology	Mandible		Middle third		Combined percentage
	No of fractures (M/F)	Percentage	No of fractures	Percentage	
RTA	536 (464/72)	68.7%%	244	31.3%%	74%
Fall	119 (56/63)	72.6%	45	27.4%	15.5%
Assault	48 (46/2)	84.2%	9	15.8%	5.4%
Sports	7 (7/0)	87.5%	1	12.5%	0.75%
Misc.	30 (27/3)	66.6%	15	33.3%	4.2%

Data with regard to associated injuries in patients with maxillofacial fractures demonstrated that patients had associated injuries. Of these 115 patients (76.66%) had head injuries and 35 patients (23.33%) had orthopedic injuries. (Table 17)

**Table 17:** Associated injuries

Associated Injuries	Number of patients	Percentage
Orthopaedic	35	23.33%
Head injury	115	76.66%
Thoracic	0	0%
Abdominal	0	0%

X –rays were advised to confirm the diagnosis of the fractures. OPG was commonly advised (71.3%) followed by IOPA (10.4%). SMV and PNS were done in 8.4% and 6.6% patients respectively. CT scan was done in 2.2 % patients and PA mandible in 1%.(Table 18)

**Table 18:** X- ray advised

X- ray	Number	Percentage
OPG	709	71.3%
PNS	66	6.6%
SMV	84	8.4%
PA mandible	10	1%
IOPA	104	10.4%
CT scan	22	2.2%

With regard to treatment modalities, 36.8% of all the mandibular fractures (740) were treated by closed reduction, 62.6% were treated using open reduction and 0.5% was under observation only. Among closed reduction group, 35.5% were treated using arch bars and other forms of interdental wiring while splints with circummandibular wiring were used in1.35%.(Table 19)

**Table 19:** Treatment modalities for mandibular fractures

Treatment modality	No. of fractures	Percentage
Closed reduction	273	36.8%
• Arch bars/wiring	263	35.5%
• Splint with circummandibular wiring	10	1.3%
Open reduction		
• Bone plates	463	62.6%
Observation only	4	0.5%
Total	740	100%

Of the 314 middle third fractures, 48.4% were treated using closed reduction, 39.5% using open reduction, 12.1% using observation only. Among closed reduction group, arch bars and other forms of interdental wiring were used in 37.9%, splints in 1.2%, internal skeletal suspension in 3.8% and Gillie’s temporal approach in 5.4%.(Table 20)

**Table 20:** Treatment modalities for middle third fractures

Treatment modality	Number of fractures	Percentage
Closed reduction	152	48.4%
• Arch bars/wiring	119	37.9%
• Splint	4	1.2%
• Internal skeletal suspension	12	3.8%
• Gillie’s temporal	17	5.4%
Open reduction		
• Bone plates	124	39.5%
Observation only	38	12.1%
Total	314	100%

## Statistical analysis

Pearson Chi-Square test was used to evaluate the association/significance between the different variables/parameters. 

The association between the time of injury and incidence of trauma was highly significant (P=0.019; p<0.01) and between incidence of trauma and mechanism of injury was very highly significant (p<0.001).

The association between incidence of trauma and alcohol consumption was also significant (p=0.048; p<0.05).

Very high significance was observed when age was associated to mechanism of injury, type of passenger, alcohol consumption and incidence of trauma (p<0.001).

No significant association was found between the incidence of trauma to the type of vehicle or (p>0.05) and the mechanism of injury to helmet use (p>0.05).

## Discussion

This study showed that the maxillofacial fractures predominantly occurred in the age group of 21-30 years (38.3%), followed by 31-40 years (20.2%) and 11-20 years (19.7%). These findings being similar with the previous studies. ^8,9,10,11,12^ The high incidence in 3rd decade of life might be due to the facts that people belonging to this decade are more active, energetic, take active participation in dangerous exercises and sports activities and mostly involved in violence. Men aged 21-40 years represent a group with intense social interaction and higher rates of morbidity, making them more susceptible to transport accidents and interpersonal violence.^13^

In the age group 0-10 years, incidence of the maxillofacial fractures was 6% in the present study. This finding was close to some previous studies, one of which showed an incidence of 9%,^14^ and 12% incidence^15^ was reported in the other. The low incidence has been explained by the high elasticity of children’s bones, the smaller face relative to head size and a decreased exposure to major trauma. ^16^

An incidence of 1.3% was noted for geriatric ( >60 years) maxillofacial fractures in this study, probably as this age group is less active and less involved in outdoor activities. Similar incidence was found by Kadkhodaie MH in Iran ^17^ and Mahmeed BEA in Kuwait.^18^

In men as compared to women the incidence of maxillofacial fractures had a ratio of 4.2: 1. This can be explained by the fact that men are more involved in outdoor activities and are also exposed to violent interactions as compared to females who are less exposed due to social and religious limitations. Male vehicle drivers also far outnumber females. ^15^ Similar ratio of 4:1 has been found in Finland by Salonem EM in 2010. ^19^

The analysis of the data on patients in the present study in reference to the time of the day exhibited that the most of accidents occurred at night (48.1%). Active nightlife and increasing number of clubs and pubs result in increase in traffic during night. Other reasons may be headlights glare at night and people are more drowsy and sleepy while returning late night from their jobs.

In our study considering the mechanism of injury, 49.9% of patients were moving at the time of injury and head on collision attributed to 40.8% of road traffic accidents, which was in accordance with a study conducted in Nigeria. ^20^ Head-on collision is at its greatest on roads with narrow lanes, sharp curves, and no separation of lanes of opposing traffic and high volumes of traffic.

According to this study, 71.89% maxillofacial fractures were caused by road traffic accidents followed by falls (16.2%) and assaults (5.6%). Road traffic accidents are the main cause of maxillofacial trauma. ^21,22,23^ The reasons for higher frequency of RTA in developing countries are inadequate road safety awareness, unsuitable road conditions without expansion of the motorway network, violation of speed limit, old vehicles without safety features, not wearing seat belts or helmets, violation of highway code and use of alcohol or other intoxicating agents. ^22^

Two wheelers were responsible for the majority of road traffic accidents in the present study (66.2% of road crashes), probably because two wheelers are very popular as a mode of transport due to their fuel efficiency and ease of use in congested traffic.^24^

Only 10% people riding on two wheelers were wearing helmets in our study. In developing countries people avoid using safety measures. The frequency of wearing helmet in Tehran is 8.6%,^25^ Vietnam 29.94% ^26^ and in Greece 20.02%.^27^

In the present study, most of the patients injured in RTA are in Group III i.e. 21-30 years. These findings being similar with the previous studies. ^28,10^ This is due to the reason that people of this age group are inexperienced drivers; they are most likely to exceed speed limits and do not use proper safety measures. Of the total number of patients included in the study there were 464 male patients and 72 female patients, who reported with a history of RTA.

Fall from height was the second most common cause of maxillofacial trauma in this study, found in 16.2 % cases. This is similar to the study by Taiseer Al-Khateeb^8^ who reported 20% incidence of maxillofacial injuries due to fall. Out of the total 119 patients who underwent trauma because of fall from height, 63 were females.

Sports related maxillofacial fractures occurred in 2.8% cases in this series. All the patients in this group belonged to 1st to 4th decade of life. This can be attributed to higher interest in sports in early childhood and young age. ^29^

**Figure 1:**
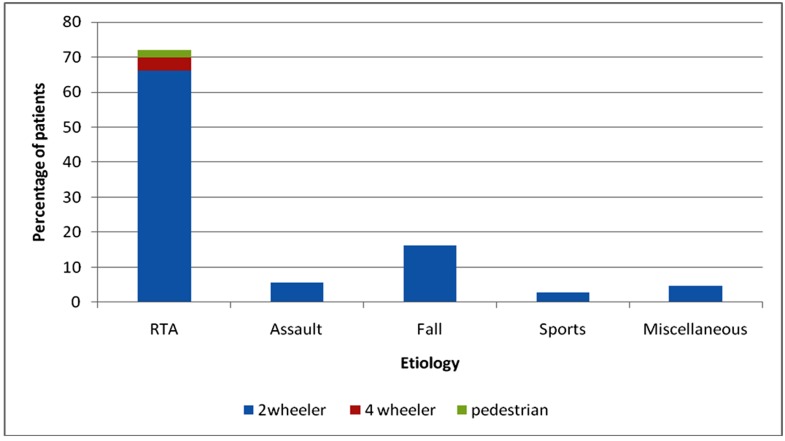
Etiology of fracture

**Figure 2:**
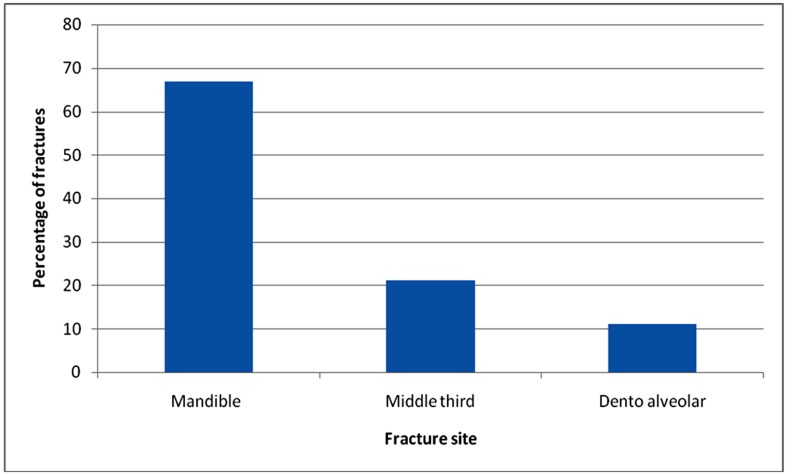
Sites of fracture

**Figure 3:**
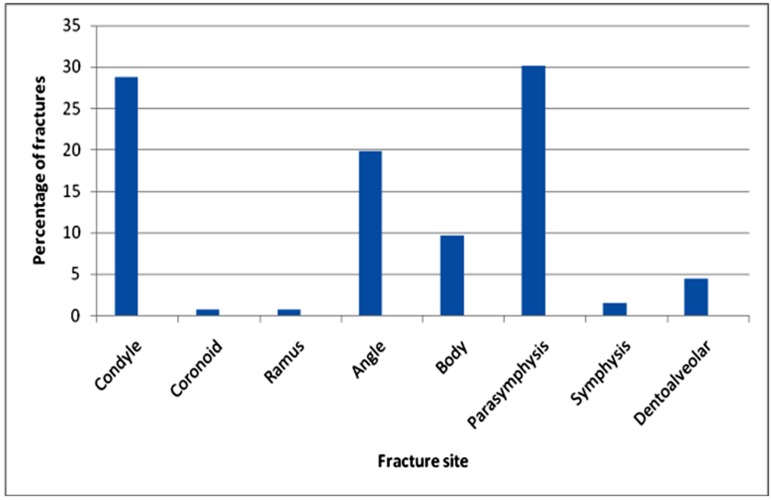
Fracture distribution in mandible

**Figure 4:**
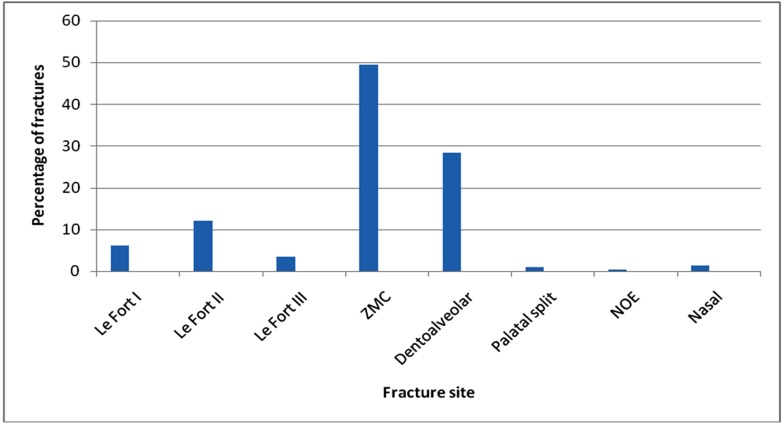
Fracture distribution in middle third

According to the site of fracture, in 70.2% cases mandible was involved as compared with 29.8% of middle third of facial skeleton fractures in this series. These results are similar to the previous studies in Jordan,^8^ UAE, ^15^ Bulgaria^30^ and Tanzania^23^ where mandible was more involved than the middle third.(Figure 1,2,3,4) 

**Figure 5:**
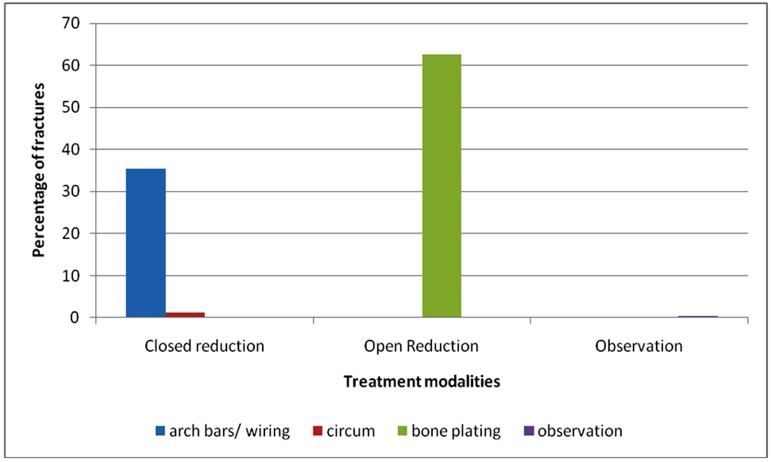
Treatment modalities for mandibular fractures

**Figure 6:**
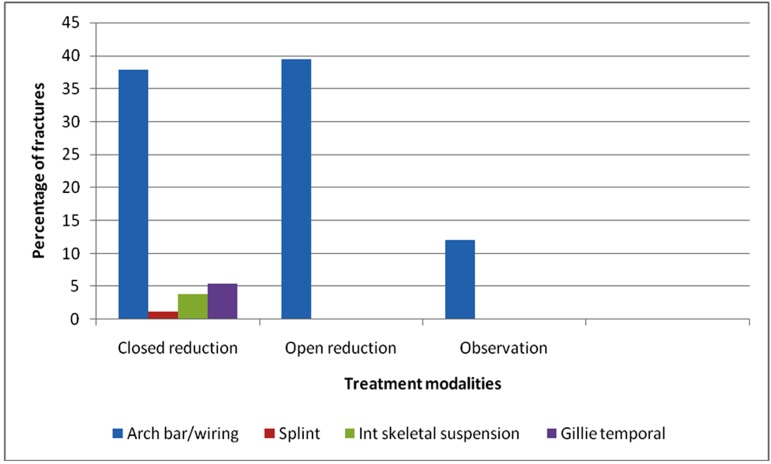
Treatment modalities for middle third fractures

Mandibular fractures (68.7%) accounted for highest number of fractures due to RTA followed by middle third fractures (31.3%) in our study. Similarly in sports related injuries , mandibular fractures (87.5%) were more common than the middle third fractures (12.5%)Mandible, being the most prominent bone in face, is often fractured more than the strongly supported middle third of the face. ^16^ (Figure 5,6)

In the present series, among the mandibular fracture sites, parasymphysis (30.2%) was the most common fracture site followed by the condyle (28.78%). The location of fracture site appears to be directly related to the cause of injury in some instances and probably reflects the direction from which force was applied to the mandible.^15^ Sports and altercation injuries most frequently resulted in angle fractures. Vehicular accidents and falls resulted in greater number of parasymphysis and condylar fractures as traffic accident victims commonly suffer posteriorly directed force to the mandible as a result of fall or chin striking the steering wheel or dashboard. ^31^

In our study, in middle third fractures, ZMC (49.4%) was most commonly involved. This is because of the prominent positions; zygomatic bone and nasal bone are more vulnerable to trauma. However in our study, there was less involvement of nasal fractures (1.3%). This was may be due to the reason that patients with nasal fractures often seek advice from ENT specialist rather than maxillofacial surgeons.

The incidence of associated injuries in the present study was 20.3%. Most common associated injury noted in our study was head injury (76.66%). This was similar to UAE studies in which associated injuries were 22.2% and with Nigerian series which reported 23% of associated injuries. ^32,23^ However more associated injuries recorded by Al-Khateeb et al in UAE (41%) ^10^ and Schaftenaar E et al in Netherland (51.5%) ^23^ were attributed to the severity of trauma. 

Out of 740 patients, 116 (15.68%) patients gave history of loss of consciousness and CT scan was done in 2.2% of patients in the present series.

Before reporting to our institute, 79.5% patients in our study took first aid from a local practioner or civil hospital. In our study most of the patients (99.6%) having maxillofacial trauma had history of bleeding (oral-89.7%, nasal-7.9% and ear-1.9%). Patients have a tendency to rush to a nearest doctor to get the bleeding arrested.

Several methods of closed reduction were used in the treatment of mandibular fractures such as Ehrich’s arch bar, other interdental wirings and splints. Out of 740 mandibular fractures, 273 (36.8%) and out of 314 mid-face fractures, 152 (48.4%) were treated with closed reduction in our study. No complications concerning occlusion and mouth opening were encountered in these patients. In developing countries people prefer closed reduction than open reduction. ^15^

In the past 15 years, plate osteosynthesis has become popular in the management of facial fractures and in the treatment of mandibular fractures.^33^ Surgeons prefer it because it offers stable and precise anatomical reduction of fragments, allows immediate recovery of function as it has no IMF, shortens the period of bone healing and decreases the recovery period. Despite the obvious advantages, it has not become popular in many developing countries mainly because of cost factors. However, 55.7% of all maxillofacial fractures in our series were treated with open reduction and internal fixation. A higher proportion of fractured mandibles were treated surgically (62.6%) than were middle third (39.5%). Routinely, patients treated with ORIF were placed in inter maxillary fixation only intra-operatively. IMF was then released in all except for the cases which had concomitant condylar fractures, planned to treat conservatively with arch bars and IMF.

In our institute, open reduction and internal fixation using miniplates is the most preferred treatment plan for maxillofacial fractures. The technical and functional advantages of miniplate osteosynthesis over maxillomandibular fixation including the ease of use, precise anatomical reduction, limited or complete avoidance of maxillomandibular fixation, functional stability and improved mouth opening have made it more preferable.

## Conclusion

Unlike in most developed countries where assaults have replaced road traffic crashes as the major cause of the injuries, in India no apparent shift from road traffic crashes as the leading cause of maxillofacial injuries was observed. Injuries have causes; they do not simply befall us from fate or bad luck. Since no magic pill is envisaged for the prevention of road traffic crashes, we need to take good stock of all the tools at our disposal, and to get down to what the developed nations have done to reduce road traffic crashes. Therefore, an awareness campaign to educate the public about the importance of restraints and protective seatbelts in cars and motorcycles should be championed. These findings should also alert the authorities, particularly the government and the Road Safety Commission to the need for the provision of good roads and traffic guidance like traffic lights at crossing junctions, enforcement of existing traffic laws regarding the mandatory use of helmets/seat belts and drink–driving legislation, and general improvement of socioeconomic conditions of the population.
